# Growth, chamber building rate and reproduction time of *Palaeonummulites venosus* (Foraminifera) under natural conditions

**DOI:** 10.1007/s00338-017-1601-x

**Published:** 2017-07-04

**Authors:** Shunichi Kinoshita, Wolfgang Eder, Julia Wöger, Johann Hohenegger, Antonino Briguglio

**Affiliations:** 1 Department of Palaeontology, University of Vienna, UZA II Geozentrum, Althanstrasse 14, 1090 Vienna, Austria; 2 Department of Petroleum Geoscience, Universiti Brunei Darussalam, Jalan Tungku Link, Gadong, BE1410 Brunei Darussalam

**Keywords:** Larger Foraminifera, Reproduction, Individual growth, Longevity

## Abstract

We investigated the symbiont-bearing benthic foraminifer *Palaeonummulites venosus* to determine the chamber building rate (CBR), test diameter increase rate (DIR), reproduction time and longevity using the ‘natural laboratory’ approach. This is based on the decomposition of monthly obtained frequency distributions of chamber number and test diameter into normally distributed components. Test measurements were taken using MicroCT. The shift of the mean and standard deviation of component parameters during the 15-month investigation period was used to calculate Michaelis–Menten functions applied to estimate the averaged CBR and DIR under natural conditions. The individual dates of birth were estimated using the inverse averaged CBR and the inverse DIR fitted by the individual chamber number or the individual test diameter at the sampling date. Distributions of frequencies and densities (i.e., frequency divided by sediment weight) based on both CBR and DIR revealed continuous reproduction throughout the year with two peaks, a stronger one in June determined as the onset of the summer generation (generation 1) and a weaker one in November determined as the onset of the winter generation (generation 2). This reproduction scheme explains the presence of small and large specimens in the same sample. Longevity, calculated as the maximum difference in days between the individual’s birth date and the sampling date, is approximately 1.5 yr, an estimation obtained by using both CBR and DIR.

**Electronic supplementary material:**

The online version of this article (doi:10.1007/s00338-017-1601-x) contains supplementary material, which is available to authorized users.

## Introduction

*Palaeonummulites venosus* (Fichtel and Moll) belongs to the group of symbiont-bearing larger benthic Foraminifera (LBF) (e.g., Hohenegger et al. 2000 and references therein). These Foraminifera can be the main carbonate producers in reef moats (Yamanouchi 1998), lagoons (Ujiie and Ono 1995) and deeper slopes (Ujiie and Shioya 1980) in the surrounding of coral reefs (Langer et al. 1997). Members of the genus *Nummulites*, the closest relative to *Palaeonummulites*, constructed large carbonate buildups in the geological past, especially during the Eocene (Racey 2001 and references therein). LBFs have complex calcium carbonate tests and prefer oligotrophic shallow-water tropical and warm temperate seas (e.g., Hallock 1999). Individuals of *P. venosus* are characterized by multi-chambered tests with involute, planispirally arranged chambers following a weak logarithmic spiral (Hohenegger et al. 2000). The hyaline-transparent, perforate walls enable life in the upper sublittoral at light intensities from 100 to 750 μmol s^−1^ m^−2^ PAR (Hohenegger et al. 2000; Wöger et al. 2016). This corresponds to water depths between 35 and 70 m in clear ocean water with an attenuation coefficient of 0.04 (Hohenegger et al. 2000; Hohenegger 2004). With its thick lenticular tests, *P. venosus* lives on sandy substrates, digging slightly into the sediment and thus resisting entrainment by strong hydrodynamics (Hohenegger 2004; Yordanova and Hohenegger 2007; Briguglio et al. 2017). A trimorphic life cycle has not been observed (Dettmering et al. 1998), and the alternation between agamonts and gamonts is caused by hindering the evacuation of gametes after gamogony by weak hydrodynamics at water depths below 50 m (Eder et al. 2017). Growth of *P. venosus* has been investigated in laboratory cultures (Krüger 1994), and, based on these investigations, chamber building rates (CBR) were calculated to estimate time-dependent oscillations in chamber growth using MicroCT. Cycles with period lengths of 14.6 and 29.2 d (Hohenegger and Briguglio 2014) and 15.22, 30.56 and 92.3 d (Briguglio and Hohenegger 2014) could be interpreted as tidal and lunar cycles. These results are potentially biased because the CBRs were based on cultures. To obtain reliable results based on CBR, these cycles must be calculated under natural conditions.

Reproduction period, longevity and CBR of LBF are important for population dynamics studies and to examine the effect of seasonal or instantaneous changes in environmental factors on growth. Population dynamics under natural conditions are easily studied in the eulittoral and uppermost sublittoral (Hallock 1974; Zohary et al. 1980; Sakai and Nishihira 1981; Fujita et al. 2000; Hohenegger 2006; Hohenegger et al. 2014). These investigations are more complicated in the deeper sublittoral due to the sampling procedure, environmental conditions (e.g., strong winds affecting the far offshore) and the stable fixing of sampling stations necessary for obtaining unbiased comparable results during the investigation period. Because of these difficulties, asexual reproduction and growth have only been investigated for deeper sublittoral LBF under laboratory conditions. Only few analyses under laboratory conditions have lasted longer than 3 months, the minimum time necessary for getting information about life expectancy (Wöger et al. 2016). Long-term growth studies in laboratory cultures approaching natural conditions at best resulted in the longest survival time of 12 months for *Heterostegina depressa* (Krüger 1994), 12 months for *Cycloclypeus carpenteri* (Lietz 1996) and 8 months for *Amphistegina lessonii* (Dettmering 1997), but this last may be truncated due to restricted observation time. A clone of *P. venosus* gamonts cultured by Krüger (1994) had longevities between 468 and 569 d when producing triflagellate gametes (Röttger et al. 1998).

To estimate reproduction time, growth and longevity of LBF under natural conditions in the sublittoral, the ‘natural laboratory’ approach has been developed (Hohenegger et al. 2014). We apply it in this study for *P. venosus*.

## Materials and methods

### Sampling and measuring

**Fig. 1:**
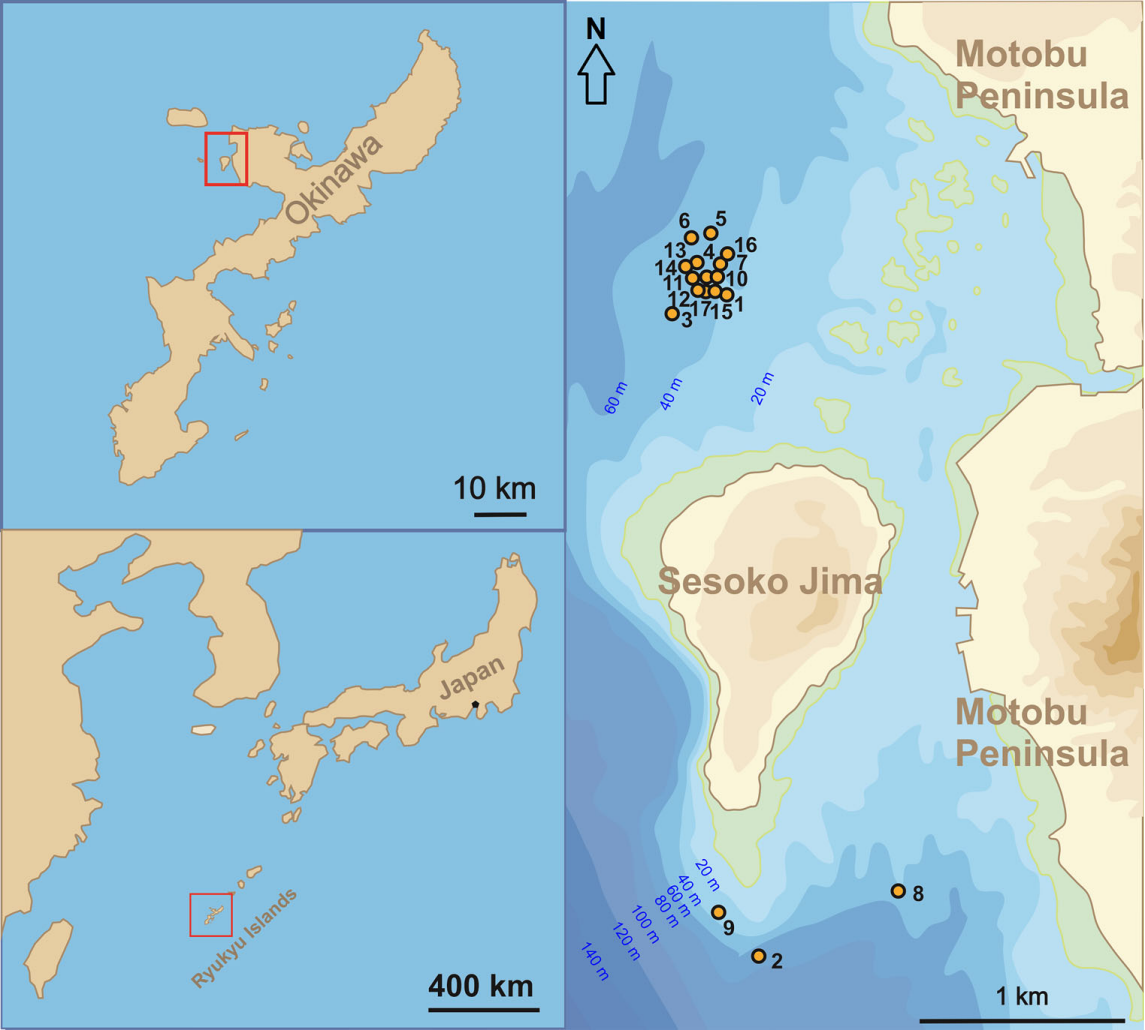
Location of study areas in Sesoko Island (Motobu, Kunigami District, Okinawa, Japan)

The investigation area is located northwest and south of Sesoko Island (Motobu, Kunigami District, Okinawa, Japan) (Table 1; Fig. 1). The northwestern sampling stations were preferred because of the more diverse LBF fauna living there on coarse sand and rubble (Hohenegger et al. 1999; Yordanova and Hohenegger 2002). Sometimes, strong winds hindering sampling in the northwest necessitated back-up sampling at the southern area, which is protected from strong winds (Fig. 1). Due to this protection, the sediment inhabited by *P. venosus* is finer in the southern area compared to the northwest stations, i.e., fine sand and silt predominate at 50 m (Ujiie and Shioya 1980).

**Table 1:** Parameters of sampling stations containing living *P. venosus*

Sample	Date	Longitude	Latitude	Depth	Temperature	Salinity	pH	Sediment		Number of individuals	
								Main grain size	Weight (g)	Agamonts	Gamonts/schizonts
1	23.04.2014	127°51.388′	26°40.086′	56.0	22.7			Coarse sand	714.6		25
2	02.05.2014	127°52.243′	26°37.126′	46.0	22.3			Fine sand/silt	381.8		33
3	09.05.2014	127°51.331′	26°40.039′	50.0	21.8		7.9	Coarse sand	1183.0		15
4	30.05.2014	127°51.5160′	26°40.220′	54.0	23.3		7.9	Coarse sand	216.2		2
5	18.07.2014	127°51.5324′	26°40.4240′	57.5	23.6		8.0	Coarse sand	999.0		31
6	19.08.2014	127°51.4673′	26°40.4231′	56.0	26.2			Coarse sand	349.5	1	3
7	10.09.2014	127°51.5281′	26°40.2410′	54.0	27.2			Coarse sand	797.2		14
8	03.10.2014	127°52.2624′	26°37.4250′	41.0	26.9	51.3		Fine sand/silt	1376.8		35
9	10.11.2014	127°51.4629′	26°37.3511′	41.0	24.7	51.2		Coarse sand	1572.8		10
10	11.12.2014	127°51.517′	26°40.218′	47.0	23.5	51.5		Coarse sand	515.1		12
11	16.01.2015	127°51.5101′	26°40.2142′	53.7	21.0	51.7		Coarse sand	309.3		18
12	13.02.2015	127°51.5076′	26°40.1711′	57.0	20.1	51.7		Coarse sand	488.4	1	7
13	04.03.2015	127°51.4727′	26°40.2670′	57.0	22.0	50.7		Coarse sand	1055.4	1	31
14	15.04.2015	127°51.4540′	26°40.2362′	58.0	23.5	51.6	8.3	Coarse sand	505.6		12
15	18.05.2015	127°51.5099′	26°40.2756′	55.0	22.9	52.3	8.0	Coarse sand	267.1	2	12
16	11.06.2015	127°51.6201′	26°40.3148′	56.5	24.0	51.4		Coarse sand	573.5		11
17	14.07.2015	127°51.5144′	26°40.1600′	50.0	27.4	51.2		Coarse sand	229.1	1	5

Samples were taken following the methods explained in Hohenegger et al. (2014), requiring at least monthly sampling intervals. Two depths were sampled by SCUBA on each date, one at ~20 m and the other at ~50 m water depth for investigating the nummulitids *Heterostegina depressa*, *Operculina ammonoides* and *P. venosus*. Because *P. venosus* lives below 40 m in the investigation area (Hohenegger 2000, 2004), only the ~50 m samples are important for analyzing reproduction and growth in *P. venosus*.

Sampling started on 23 April 2014, and continued until 14 July 2015. The required monthly sampling intervals (Hohenegger et al. 2014) could not be always maintained due to occasional bad weather conditions (e.g., winter winds from the NW, tropical cyclones) resulting in differing sampling intervals (Table 1). Four benthic surface samples were taken with a plastic box at both 20 and 50 m depth and transported to the laboratory. After 24 h resting in large trays covered with seawater, living individuals could be easily identified by their colored protoplasm completely filling the test. Living individuals were collected and identified, and—after separating specimens for growth investigations under laboratory conditions (Wöger et al. 2016)—the remaining tests were washed in fresh water and dried. After rinsing the remaining sediment in fresh water and drying, it was weighed to estimate species densities (Hohenegger et al. 2014).

Two characteristics measured on each specimen enable calculation of time-related growth, based on populations. The first is the number of chambers, which characterizes the individual growth stage (Hohenegger and Briguglio 2014). The second is the test diameter of each specimen. This characteristic was commonly used for growth determination (Röttger et al. 1984; Krüger 1994; Krüger et al. 1997) before X-ray investigations (Hohenegger et al. 2000; Yordanova and Hohenegger 2004) and MicroCT studies were possible. These diameter values were compared with the results obtained by counting the chambers. Measurements of both characteristics were taken on virtual test reconstructions obtained by MicroCT (Briguglio et al. 2011, 2013; Schmidt et al. 2013; Briguglio and Hohenegger 2014). For this purpose, the MicroCT (high-energy MicroCT Skyscan 1173, resolution 9.98 μm, source voltage 100 kV, source current 80 μA, rotation step 0.20°) of the Department of Palaeontology, University of Vienna was used. Test reconstruction was performed with the program InstaRecon (version 1.3.8.5) and Amira 5.5.0 VSG.

### Statistical methods

Only megalospheres (gamonts or schizonts) were used due to the extremely small numbers of agamonts, whose much larger numbers of chambers and test size hindered separate frequency calculations. The calculations for estimating CBRs and test growth for megalospheres are described in detail by Hohenegger et al. (2014). In brief, chambers are counted by natural numbers starting with the first chamber after the nepiont (proloculus + deuteroloculus). Test diameters are measured starting with the largest diameter of the nepiont; this follows a nonlinear (logarithmic) growth, which leads to skewed normal distributions. The natural logarithms of test size transform them to symmetrical normal distributions (Hohenegger et al. 2014).

**Fig. 2:**
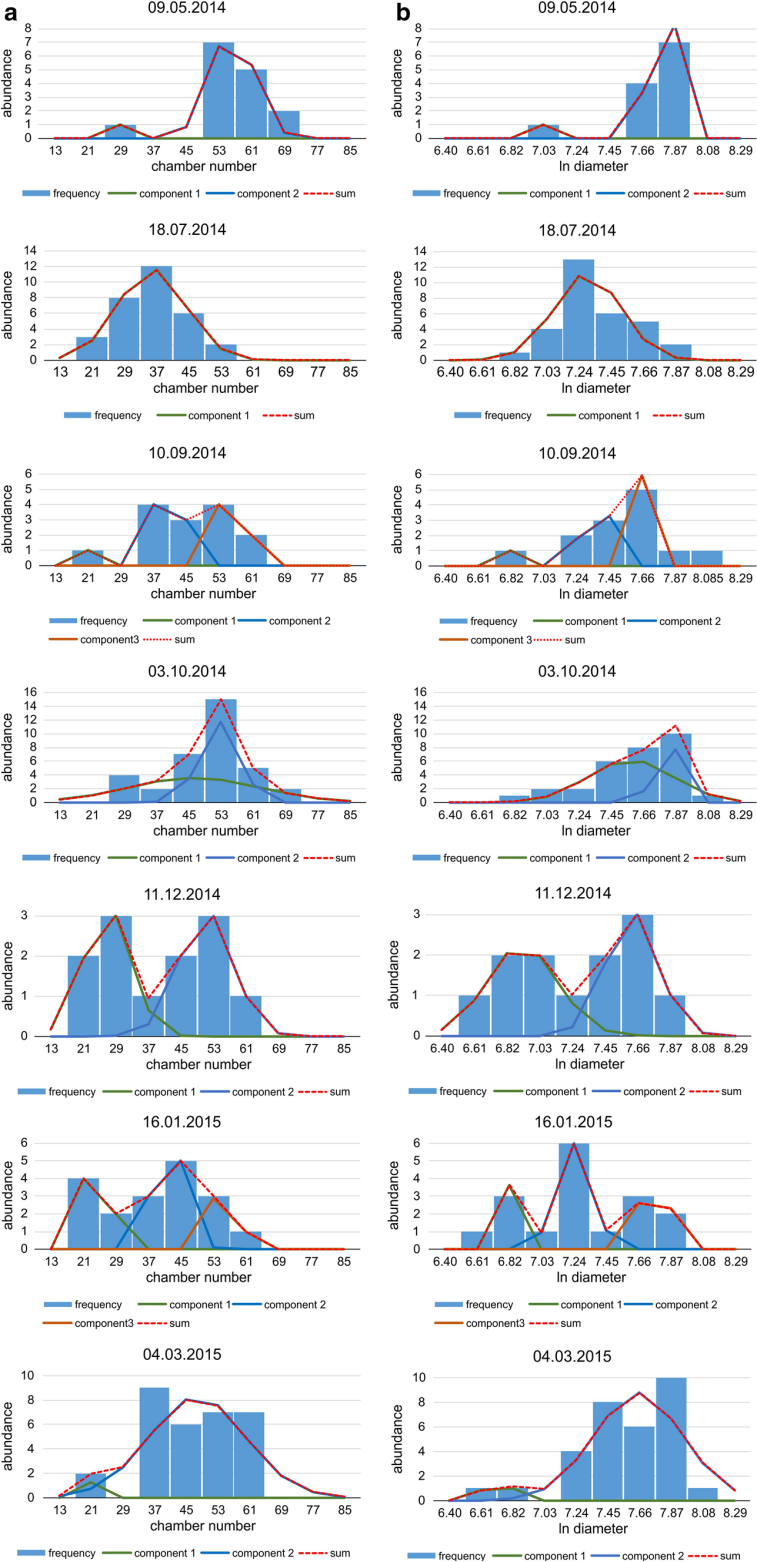
Decomposition of frequency distributions into normally distributed components **a** based on chamber number, **b** based on test diameter

Frequency diagrams were generated for the 17 sampling dates (Table 1) using identical intervals on the *x* axis (Fig. 2). The use of densities (frequency per sediment weight) as proposed in Hohenegger et al. (2014) is important for population dynamics (Hohenegger 2006) but was difficult due to sampling in different regions (NW, S). The regions had dissimilar environmental conditions at 50 m depth, expressed in different sediment composition. Nonetheless, the mean and standard deviation, necessary for calculating growth in test size and CBR, do not change using either frequencies, densities or percentages (proportions). Thus, absolute frequencies can be used without biasing these distribution parameters.

Frequency distributions were tested for normality using Chi-square tests. In case of significant deviation from normal distribution, frequency distributions were decomposed into normally distributed components using nonlinear regression based on numerical mathematics (IBM SPSS Statistics 22; Fig. 2; Table 2).

**Table 2:** Parameters of the normally distributed components after decomposition of the frequency distribution

Sample	Date	Number of chambers						Test diameter					
		Component 1		Component 2		Component 3		Component 1		Component 2		Component 3	
		Mean1	SD1	Mean2	SD2	Mean3	SD3	Mean1	SD1	Mean2	SD2	Mean3	SD3
3	09.05.2014	27.65	1.25	56.24	5.24			7.03	0.03	7.77	0.05		
5	18.07.2014	35.81	8.46					7.29	0.22				
7	10.09.2014	19.46	1.17	40.82	2.23	56.50	2.39	6.81	0.04	7.36	0.07	7.66	0.04
8	03.10.2014	32.36	2.24	52.35	6.00			7.57	0.28	7.78	0.05		
10	11.12.2014	26.78	5.70	51.15	6.53			6.91	0.22	7.62	0.17		
11	16.01.2015	24.57	2.20	41.94	3.84	56.22	2.43	6.85	0.04	7.24	0.11	7.76	0.06
13	04.03.2015	21.25	0.40	47.88	12.18			6.71	0.06	7.65	0.29		

*SD* standard deviation

Means and standard deviations of each component *j* can be used to estimate the maximum chamber number or the logarithm of test diameter at time *t* by

1 $$ m_{jt} = \bar{x}_{jt} + 3s_{jt}^{*} $$

**Fig. 3:**
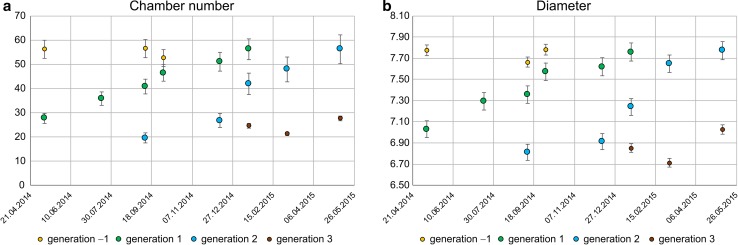
Mean (*dots*) and standard deviation *s* (*bars*) of chamber number (**a**) and test diameter (**b**) at different sampling times

Normalized standard deviations *s* ^*^ are calculated using the mean of the coefficients of variance (CV) and recalculating the appropriate standard deviation by

2 $$ s_{jt}^{*} = {\text{CV}}_{\text{mean}} /\bar{x}_{jt} $$

(details in Hohenegger et al. 2014). The time-related dependence of components (Fig. 3) shows up to four generations within the time interval of 15 months, restricted to two generations within a year. Both generations increase continuously with time, marked by different onsets but similar tendencies. The onset of a generation is adjusted to 30 d before the date of the component with the lowest maximum *m*. The onset in chamber numbers is characterized by

$$ m_{j1} = 2\;{\text{and}}\;m_{j2} = 3, $$

as determined by growth investigations in the laboratory (Krüger 1994). For the test diameter, the mean over all investigated individuals at chamber number 2 and chamber number 3 for the first 2 d was used, resulting in

$$ m_{j1} = \ln 252\; \upmu{\text{m}} = 5.529\;{\text{and}}\;m_{j2} = \ln 263\; \upmu{\text{m}} = 5.572. $$

In the following, the CBR was calculated for each generation using the equation

3 $$ m_{jt} = m_{{j {\rm max}}} t /\left( {B + t} \right) $$

that resembles the Michaelis–Menten function running through the origin (Hohenegger et al. 2014). The parameter *B* indicates the time *t*, where $m_{{j {\rm max}}} /2$ is reached. Because the test diameter starts to increase at the nepiont (*t* = 0) with values >0, Eq. 3 must be modified to

4 $$ m_{jt} = m_{{j\,{ \hbox{max} }}} t /\left( {B + t} \right) + 5.346. $$

Student’s *t* tests were used to check the coincidence in parameters $m_{\rm max}$ and *B* in both chamber number and test diameter between the two generations with onsets within the investigated periods.

Function parameters of the generation with the higher $m_{\rm max}$ were used to estimate the date $t\left( 0 \right)$ when specimen *i* was born. Therefore, the number of chambers *n* of specimen *i* at the time $t\left( i \right)$ when the specimen was collected determines the onset time by

5 $$ t\left( 0 \right) = t\left( i \right) - n_{i} B /\left( {m_{\rm max} - n_{i} } \right). $$

This formula changes to

6 $$ t\left( 0 \right) = t\left( i \right) - \left[ {B (\ln d_{i} - 5.530)} \right] /\left( {m_{\rm max} - \ln d_{i} + 5.530} \right) $$

when the logarithm of the test diameter *d* is used.

Onset times of all investigated specimens were depicted for both chamber number and test diameter in frequency diagrams with monthly intervals. Counts of densities (${\text{count}}^{ *}$) per specimen *i* of sample *k* instead of simple counts (${\text{count}} = 1$) must be used because the latter are biased by differing sizes of samples *k,* thus necessitating the transformation

7 $$ {\text{count}}_{ik}^{*} = 1 / {\text{sample}}\;{\text{size}}_{k} . $$

In our investigation, sediment weights in kg represent sample size. Onset frequencies were then used to find periods in reproduction based on Lomb periodograms (Press et al. 1992) and compared with sinusoidal regression based on Nyquist frequencies (Shannon 1949) and harmonic series (Hammer 2016).

Calculating the reproduction time for every individual enables an estimate of longevity, even though densities are strongly biased and could not be used in the manner described by Hohenegger et al. (2014). Here, longevity can be easily estimated by calculating the maximum difference in days between the individual reproduction date and the sampling date

8 $$ \hbox{max} \left[ {t\left( i \right) - t_{i} \left( 0 \right)} \right]. $$

Complex statistical investigations used the program packages IBM SPSS Statistics 22 and PAST 3.02 (Hammer et al. 2001), while the remaining calculations were performed in Microsoft Excel.

## Results

### Chamber building rate and test diameter increase rate

The 249 megalospheres (possibly gamonts) investigated (Electronic Supplementary Material, ESM, Table S1) show a highly significant correlation between chamber number and test diameter at each sample site (Table 3). This corroborates the coincidence of results and inferences based on chamber number and test diameter.

**Table 3:** *χ* ^2^ tests for correlations between chamber number and the logarithm of test diameter (in µm) at the 17 sampling sites and for normal distribution of chamber number and the logarithm of test diameter

Date	*n*	Correlation		Chamber number		Test diameter	
		*R* ^2^	*p*(H_0_)	*χ* ^2^	*p*(H_0_)	*χ* ^2^	*p*(H_0_)
23.04.2014	24	0.7874	3.75E−09	11.13	4.21E−02	23.85	4.90E−04
02.05.2014	28	0.8601	6.56E−13	9.28	6.74E−02	7.30	9.95E−02
09.05.2014	12	0.8080	3.50E−05				
30.05.2014	2						
18.07.2014	31	0.7705	4.46E−11	8.84	7.44E−02	7.60	9.48E−02
19.08.2014	2						
10.09.2014	13	0.8816	9.92E−07	11.99	3.29E−02	32.76	1.26E−05
03.10.2014	30	0.7446	4.31E−10	27.94	9.39E−05	27.01	1.37E−04
10.11.2014	8	0.8946	1.91E−04	11.88	3.40E−02	32.70	1.29E−05
11.12.2014	12	0.9906	9.11E−12	22.57	8.08E−04	20.11	2.08E−03
16.01.2015	17	0.8523	6.42E−08	38.02	1.32E−06	67.27	2.44E−12
13.02.2015	7	0.6287	1.67E−02	0.26	7.72E−04		
04.03.2015	31	0.9033	1.50E−16	21.21	1.37E−03	106.51	2.32E−20
15.04.2015	8	0.7048	4.56E−03	26.24	1.88E−04	25.41	2.63E−04
18.05.2015	12	0.8206	2.48E−05	121.94	1.45E−23	30.69	3.01E−05
11.06.2015	8	0.8803	2.81E−04	42.28	2.04E−07	19.73	2.39E−03
14.07.2015	4	0.7538	6.59E−02	30.51	3.25E−05	1.26	2.54E−02

Note the difficulty/impossibility of checking significance in samples with *n* < 4 specimens even though high correlation is present (sample from 14 July 2015)

Frequency distributions based on chamber number and test diameter show statistically significant deviations from normal distribution in 12 monthly samples (Table 3). They demonstrate trends in component parameters through time, where up to three components can be distinguished within a single month (Fig. 2; Table 2). Decomposition into normally distributed components could be performed on seven monthly samples containing abundant specimens, thus giving a sense of the decomposition (Fig. 2).

Relating the component parameters $\bar{x}_{jt}$ and $s_{jt}$ to time (Fig. 3) reveals four different generations during the investigation period in both characteristics. Generations 1 and 2 cover large portions of the investigation period, while generation 1 is represented by large individuals with high chamber numbers from the beginning of the investigation period until September 2014. Generation 3 starts in January 2015 with small individuals characterized by low chamber numbers.

**Fig. 4:**
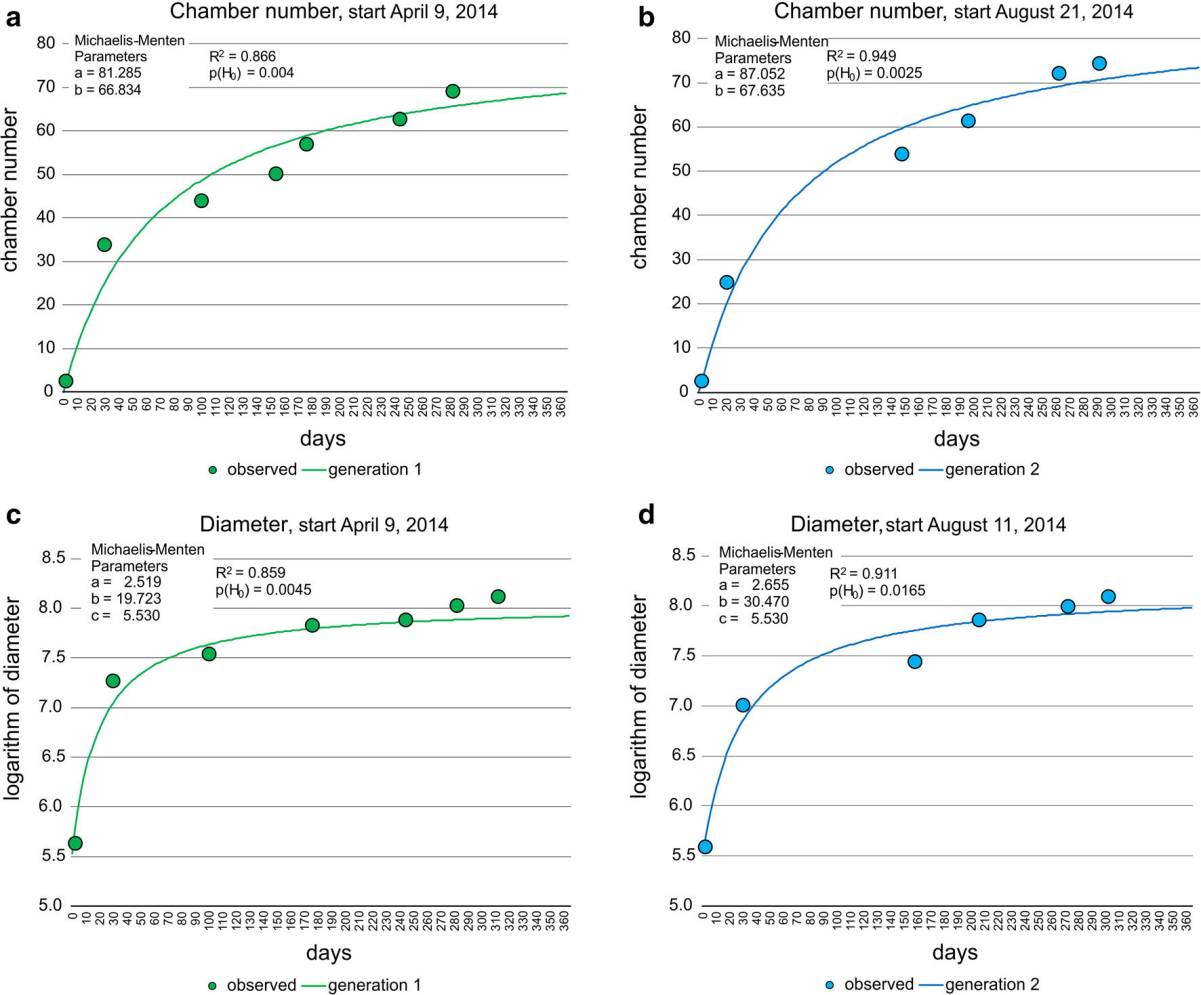
Fit of chamber building rate (CBR) and test diameter increase rate (DIR) in first and second generation by Michaelis–Menten functions, **a** CBR of the first generation, **b** CBR of second generation, **c** DIR of first generation, **d** DIR of second generation

The fit of generations 1 and 2 by the Michaelis–Menten function using the transformed means $m_{jt}$ (Eq. 1) resulted in significant fits (Fig. 4) for both chamber number and test diameter (Eqs. 3, 4). Moreover, the Michaelis–Menten function parameters *a* indicating the upper limits and *b* determining the increase of the function (low values = strong increase, high values = weak increase) do not differ significantly between generations (ESM Table S2).

Both CBRs correlate with the rate experienced in laboratory cultures (Krüger 1994), where offspring of *P. venosus* attained seven chambers within 1 week, building one chamber every day. Following the natural laboratory approach, 7.71 chambers (generation 1) and 8.16 chambers (generation 2) are built within a week, thus simulating the growth in laboratory cultures.

**Fig. 5:**
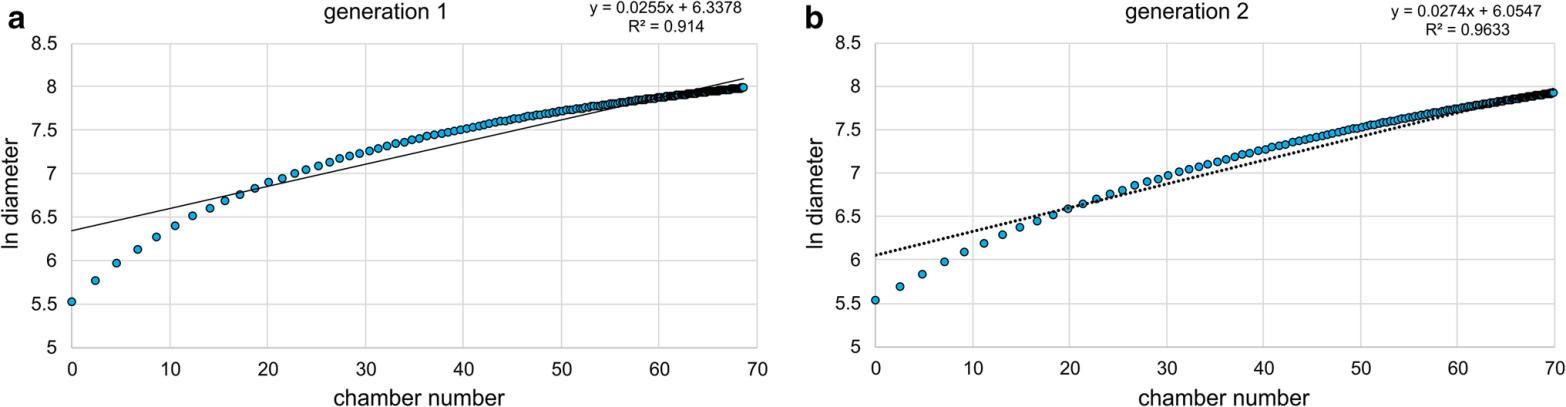
Correlation in Michaelis–Menten functions between chamber building rates (CBR) and test diameter increase rates (DIR), **a** correlation of first generation, **b** correlation of second generation

The Michaelis–Menten functions in both generations based on chamber numbers with test diameters were highly, but not linearly, correlated (Fig. 5). Deviations from linearity are mainly expressed in the initial test part (Fig. 5).

The function of the second generation with the higher maximum value *a* was used for the mean CBR, because it had a higher limit including all individuals, and the parameters of the first generation were used to estimate DIR during further procedures for the same reason.

### Estimating reproduction time

Several approaches were used to estimate the birth date for every individual. As explained above, Eq. 5 can be applied to estimate the birth date of any individual based on the CBR using the Michaelis–Menten function parameters of the second generation. The growth rate for the test diameter of the first generation was also used to estimate the individual onset date by Eq. 6.

**Fig. 6:**
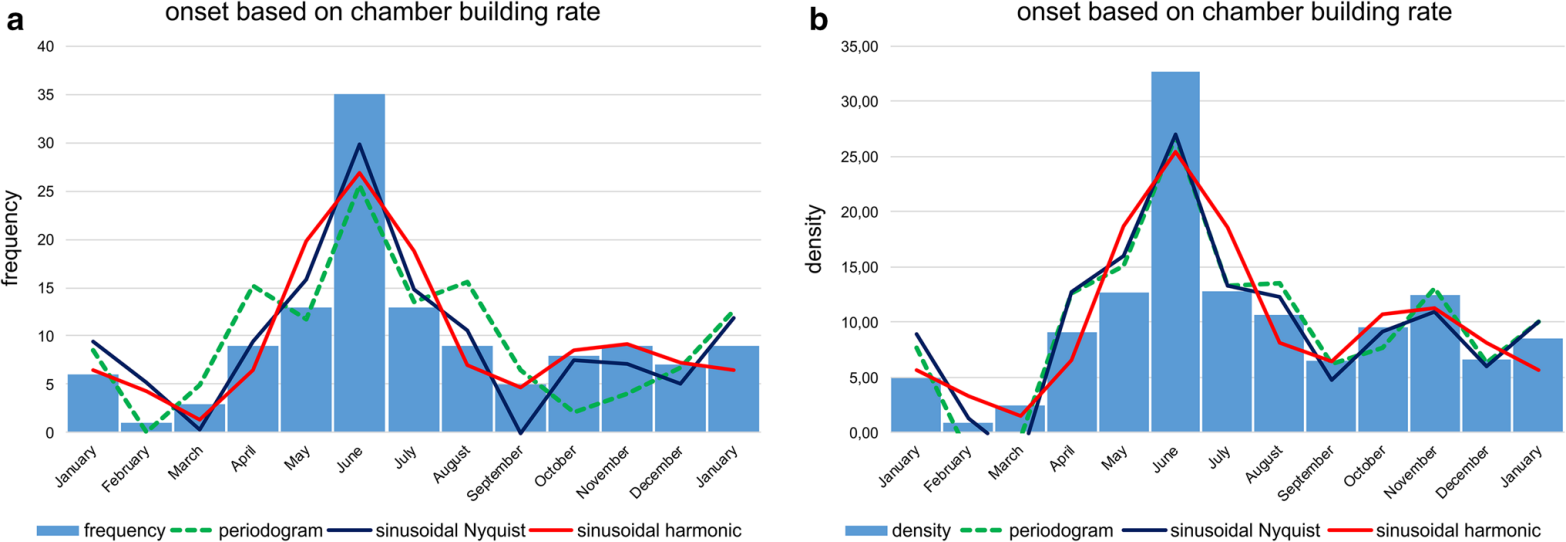
Histograms of reproduction dates using chamber building rate (CBR). Frequency histograms are based on simple counts (**a**); density histograms are based on counts standardized by sediment weight (**b**). Fit by sum of significant periods obtained by Lomb periodograms, sums of sinusoids based on Nyquist frequencies and harmonic series

Histograms with monthly intervals based on simple counts (frequencies in Figs. 6, 8) and counts normalized by sediment weight (Eq. 7; densities in Figs. 6, 8) were established to check periodicities in reproduction, on the one hand based on chamber number (Fig. 6), on the other hand based on test diameter (Fig. 8).

According to reproduction times based on CBRs, the histograms do not differ strongly between frequencies and densities: both are characterized by two identical peaks with a dominant peak in June, followed by a second, smaller peak in November (Fig. 6). The latter peak is better expressed in the histogram based on densities.

**Fig. 7:**
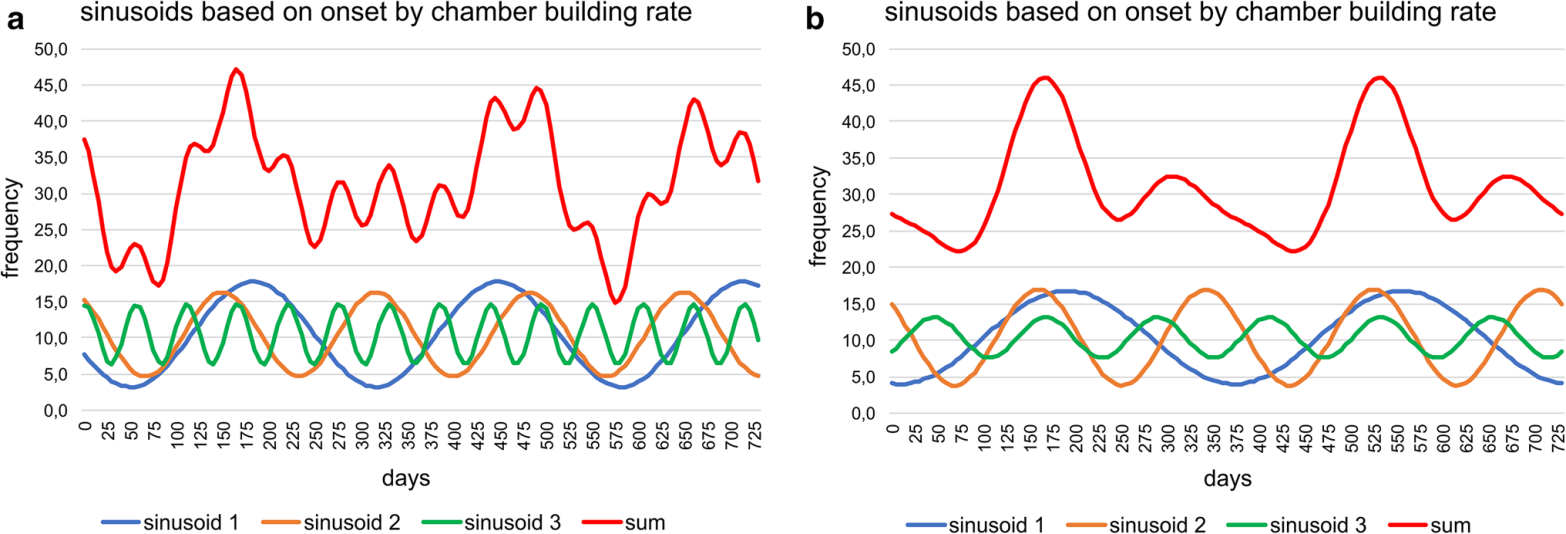
Oscillations of the reproduction dates over 2 yrs (730 d) based on chamber building rates. **a** Sums of sinusoids using Nyquist frequencies, **b** sums of sinusoids using harmonic series

Fitting histograms with a periodic function, the best fit is obtained on densities by the sum of sinusoids using Nyquist frequencies (ESM Table S3); the significant periods are 367, 179.4, 95.9 and 68.2 d and the corresponding amplitudes 5.77, 8.46, 2.97 and 5.01. The height of amplitudes marks the importance of sinusoids. Following this fitting method, the sum of sinusoids is not repeated in the succeeding year (Fig. 7a). A perfect repeat in the following year is obtained by sinusoids based on harmonic series with the significant periods 365, 182.4, 121.6 and 91.2 d and the dedicated amplitudes 5.27, 8.03, 2.46 and 1.96 (Fig. 7b). These amplitudes demonstrate the importance of the first and second sinusoid and the weak influence of the third and fourth. The fit by sinusoids using the harmonic series is slightly less significant than the fit by Nyquist frequencies, but higher than the fit by Lomb periodograms using the same number of sinusoids (ESM Table S3).

**Fig. 8:**
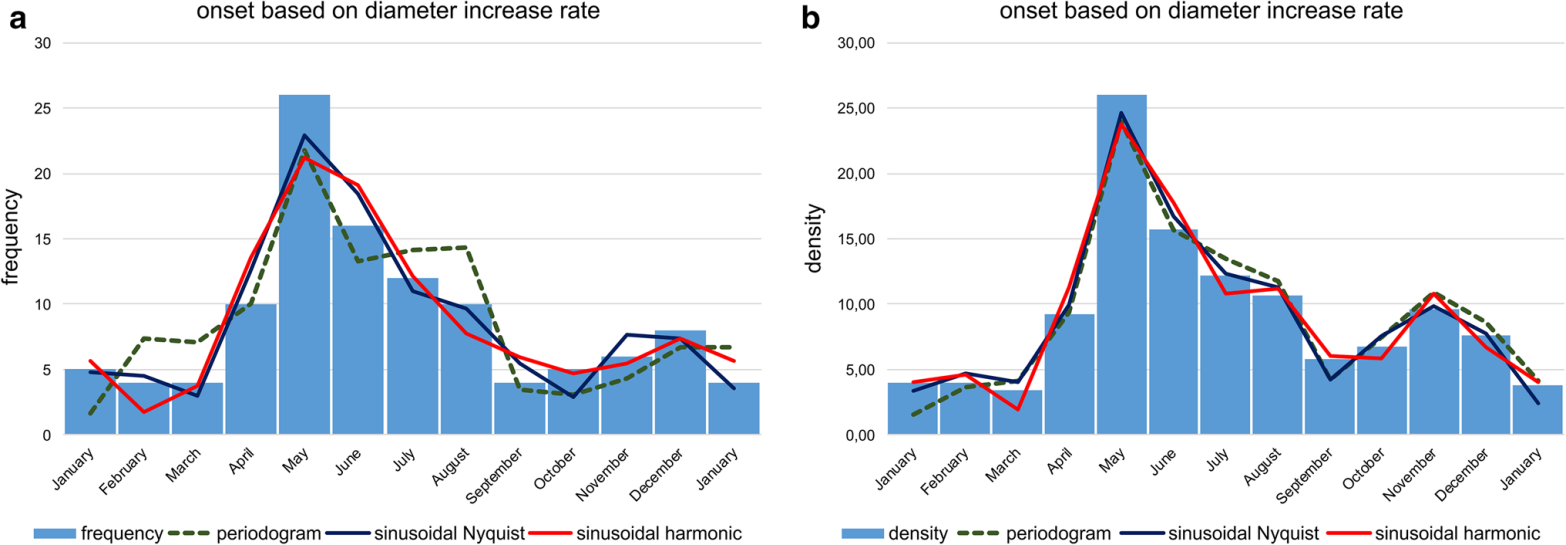
Histograms of reproduction dates using test diameter increase rate. Frequency histograms are based on simple counts (**a**); density histograms are based on counts standardized by sediment weight (**b**). Fit by sum of significant periods according to Lomb periodograms, sums of sinusoids based on Nyquist frequencies and harmonic series

Histograms for onset time based on DIR resemble histograms based on CBR. The main differences lie in the right-skewed first peaks, which are positioned in June (Fig. 8). Reproduction weakness in February is not as clearly expressed as by the CBR. Nonetheless, the second peak in November/December is more distinct than in histograms based on CBRs (Fig. 6).

**Fig. 9:**
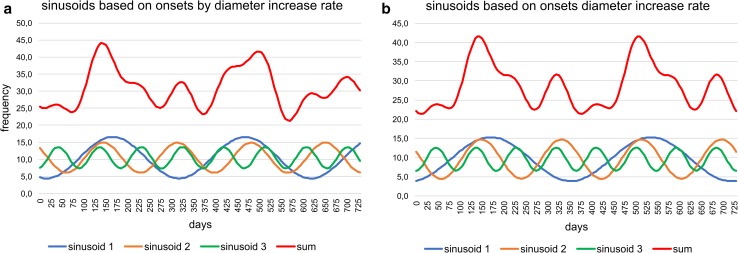
Oscillations of the reproduction dates over 2 yrs (730 d) based on test diameter increase rates. **a** Sums of sinusoids using Nyquist frequencies, **b** sums of sinusoids using harmonic series

The best fit on densities is by the sum of sinusoids using Nyquist frequencies (Fig. 8) with the main period of 187.5 d and the highest amplitude (6.55), followed by the periods of 367 and 88.7 d with similar amplitudes (2.95, 3.51). This leads to different peaks of the sum of sinusoids in the succeeding year (Fig. 9a). The significant fit by sinusoids based on harmonic series (ESM Table S3) shows the same frequencies in the following year (Fig. 9b). Here, the amplitudes of the four significant sinusoids (365, 182.4, 121.6 and 91.2 d) with their corresponding values 3.06, 6.52, 0.62 and 3.21 demonstrate the weak importance of the third sinusoid (period 121.6 d); it can therefore be neglected (Fig. 9b).

Following the density histogram based on CBR (Fig. 6), the reproduction rate is weakest in February. Between March and September, the diagram shows the highest peak in June, with a similar increase (April–May) and decrease (July–August). After a local minimum in September, the second, smaller reproduction peak is evident in November, followed by a strong decrease to February. These tendencies can be significantly fitted by the sum of sinusoids based on harmonic series (Fig. 7) and are stable over the year.

The density diagram based on DIR (Fig. 8) differs from the CBR, with the reproduction minimum in March, a strong increase to the maximum in May, followed by a slight decrease to the local minimum in September. The following smaller second peak in November is the same as identified by the CBR. In the sum of sinusoids, distribution skewness is caused by a prominent third sinusoid (Fig. 9), while the third sinusoid based on CBR is less prominent (Fig. 7).

### Estimating life expectancy

Longevity of *P. venosus* under natural conditions is understood as the maximum number of days between sampling date and estimated birth date calculated for every individual (Eq. 8). Due to the differing Michaelis–Menten functions for CBR and DIR, the estimated maxima also differ. Based on CBR, the estimated maximum is 413 d, while for DIR the maximum is 432 d, corresponding to 14 months. Thus, both estimations support the assumption that the longevity of megalospheres in *P. venosus* is over 1 yr, possibly 1.5 yr depending on the season of birth.

## Discussion

The averaged CBR and DIR of *P. venosus* gamonts were modeled by Michaelis–Menten functions, which are based on the decomposition of monthly frequency distributions into normally distributed components.

In both growth models, two generations were observed within 1 yr with statistically similar growth parameters. Therefore, both generations, either in CBR or in DIR, show the same effects due to seasonal changes in the hydrological parameters temperature, transparency and hydrodynamics, which are the most important for symbiont-bearing LBF.

The CBR estimated by the natural laboratory approach closely approximates the rate observed in laboratory cultures, where daily constructions of a single chamber were observed after the offset of the nepiont, resulting in seven chambers within a week (Krüger 1994). In our investigation, the first derivate of the CBR fitted by the Michaelis–Menten function confirms the decreasing rate in building one chamber per day, starting with a rate of 1.18 (generation 1) and 1.25 (generation 2) for the first day, continuously decreasing to the rate of 0.83 chambers (generation 1) and 0.88 chambers (generation 2) after 2 weeks (Table 4). The mean number of chambers after 7 d is 7.71 for the first and 8.16 for the second generation. These higher values of natural growth rates versus laboratory cultures (Krüger 1994) can be explained by more convenient conditions in the natural environment leading to an increased CBR (Hohenegger et al. 2014).

**Table 4:** Chamber number, chamber building rate, test diameter and rate of test diameter increase of two time-delayed generations of megalospheres of *P. venosus* within the first 2 weeks

Day	Number of chambers				Log test diameter			
	Generation 1		Generation 2		Generation 1		Generation 2	
	*y*	d*y*/d*x*	*y*	d*y*/d*x*	*y*	d*y*/d*x*	*y*	d*y*/d*x*
1	1.20	1.18	1.27	1.25	5.66	0.12	5.62	0.08
2	2.36	1.15	2.50	1.21	5.77	0.11	5.70	0.08
3	3.49	1.11	3.70	1.18	5.87	0.10	5.77	0.07
4	4.59	1.08	4.86	1.15	5.97	0.09	5.84	0.07
5	5.66	1.05	5.99	1.12	6.05	0.08	5.91	0.06
6	6.70	1.02	7.09	1.09	6.13	0.08	5.97	0.06
7	7.71	1.00	8.16	1.06	6.21	0.07	6.03	0.06
8	8.69	0.97	9.21	1.03	6.28	0.07	6.09	0.05
9	9.65	0.94	10.22	1.00	6.34	0.06	6.14	0.05
10	10.58	0.92	11.21	0.98	6.40	0.06	6.19	0.05
11	11.49	0.90	12.18	0.95	6.46	0.05	6.24	0.05
12	12.37	0.87	13.12	0.93	6.51	0.05	6.29	0.05
13	13.24	0.85	14.03	0.91	6.56	0.05	6.33	0.04
14	14.08	0.83	14.93	0.88	6.61	0.04	6.37	0.04

Test diameter growth was weaker in laboratory cultures than natural growth. In laboratory culture, the mean diameter was ~400 μm after 30 d (Krüger 1994), but under natural conditions this size was reached after only 9 d following Eq. 6. The differences between sizes in cultured versus natural populations were smaller in larger specimens with their lower growth rate. A size of 1450 μm obtained after 5 months (~150 d) in culture (Krüger 1994) was reached after 60 d under natural environmental conditions. Note that growth rates did not differ between geographically separated regions, as demonstrated by *Heterostegina depressa* from Okinawa and Hawaii, but were much lower in laboratory cultures (Eder et al. 2016). This may be caused by the impossibility of perfectly simulating natural conditions in the laboratory. This is documented in *H. depressa* by large differences in chamber size during growth under laboratory conditions, deviating from the regular increase in specimens grown in the sea (Table 1 and Fig. 4 in Röttger 1972) and by irregular growth and partial dissolution of septa (Figs. 2.2 and 2.3 in Hohenegger et al. 2014). In our case, both investigations were on individuals from Sesoko Island, thus confirming the reduced growth in laboratory cultures compared to natural conditions.

**Fig. 10:**
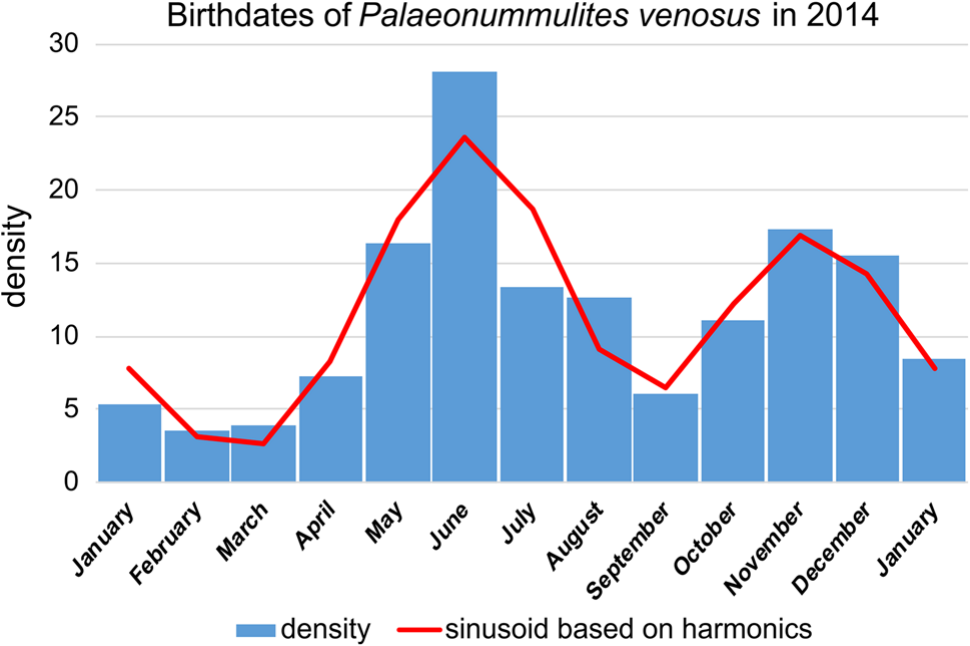
Density histogram of birth dates (*blue bars*) combining dates based on chamber number (Fig. 6b) and test diameter (Fig. 8b). Fit by sums of sinusoids (*red line*)

Frequency and density histograms of birth dates using the Michaelis–Menten functions for the averaged CBR (Eq. 5; Fig. 6) and the averaged DIR (Eq. 6; Fig. 8) give slightly different results. Both indicate reproduction throughout the year, explaining the presence of both small and large individuals in almost all samples, but with slightly different frequencies (Fig. 2). This contrasts with other reproduction studies on LBF. A single mass reproduction event is restricted to June in the porcelainous *Peneroplis antillarum* (Hohenegger et al. 2014), *Amphisorus hemprichii* (Zohary et al. 1980), the hyaline *Calcarina gaudichaudii* (Hohenegger 2006) and *Baculogypsina sphaerulata* (Sakai and Nishihira 1981; Hohenegger 2006), all studied in the subtropics. In contrast, two time-restricted events in June and November have been described for the porcelainous *Amphisorus kudakajimaensis* (Fujita et al. 2000; Hohenegger 2006). Similarities are evident with the tropical eulittoral *B. sphaerulata*, which shows constant birth rates over the year, but without reproduction peaks (Fujita et al. 2016); this seems to be characteristic for tropical in contrast to subtropical LBFs. Differences in the density histograms of reproduction onsets are caused by the nonlinear correlation between the Michaelis–Menten functions based on chamber number and test diameter (Fig. 5). Nonetheless, the main results of these investigations are that *P. venosus* has continuous reproduction over the year with a minimum in February/March, a maximum in June, a local minimum in September, and a local maximum in November (Fig. 10).

Comparison with other, non-continuously reproducing LBF shows similarities with the shallow-water porcelainous *A. kudkajimaensis*. Although the reproduction modalities are different, the time-restricted reproduction of *A. kudkajimaensis* in June with a loss of all individuals and the second reproduction in November (Fujita et al. 2000; Hohenegger 2006) coincides with the reproduction maxima in the continuously reproducing *P. venosus*.

The calculated maximum lifetime of *P. venosus* gamonts (megalospheres) of 413 d (CBR) or 432 d (DIR) leads to the assumption that the maximum longevity could be about 1.5 yr, which is the observed lifetime of a clone of gamonts cultured in the laboratory (Röttger et al. 1998). This is similar to the lifetimes of the eulittoral *C. gaudichaudii* and *B. sphaerulata* (Hohenegger 2006). Differences to the star-shaped forms can be found in the continuous reproduction rate and the presence of two main reproduction times.

Summarizing the results of the ‘natural laboratory’ approach based on the averaged CBR and test DIR of the gamont generation, the reproduction of *P. venosus* occurs throughout the year and is characterized by two peaks. The highest rate is in June; the second peak of reproduction is in November. Weakest reproduction is in February and early March. This is supported by the presence of very few agamonts in the summer and winter samples, while they were missing in other months. The reproduction peaks could be caused by the raining seasons with crossing of the monsoon front in May and September, leading to reduced transparency and higher input of inorganic nutrients (Wöger et al. 2016). This reproductive pattern is similar to the porcelainous *A. kudakajimaensis*, but differs in that reproduction is lacking between reproduction periods in *A. kudakajimaensis*. This reduces lifetime of *A. kudakajimaensis* to 1 yr for the early summer generation and to half a year for the late autumn generation (Fujita et al. 2000). Reproduction throughout the year also takes place in the hyaline eulittoral ‘star sand’ Foraminifera *B. sphaerulata* in tropical, equatorial regions, but without distinct reproduction peaks (Fujita et al. 2016). This is different to its reproduction in the subtropical NW Pacific, where reproduction is concentrated in late spring and early summer (Sakai and Nishihira 1981; Hohenegger 2006). Thus, it would be interesting to determine whether the reproduction of *P. venosus* shows similar differences between tropical and subtropical regions—in the one region without peaks and in the other with two peaks in late spring/early summer and late autumn.

The maximum lifetime of gamonts from their birth to reproduction seems to be around 1.5 yr, like the hyaline eulittoral ‘star sand’ *B. sphaerulata* (Sakai and Nishihira 1981) and *C. gaudichaudii* (Hohenegger 2006). Lifetime of agamonts could not be tested due to the extremely few individuals and differences in the CBRs but have been estimated for a maximum of 3 yr (Briguglio and Hohenegger 2014).

The ‘natural laboratory’ approach presented here can be used for other abundant, free living organisms in the surrounding of coral reefs, especially mollusks.

## Electronic supplementary material

Below is the link to the electronic supplementary material.

Supplementary material 1 (XLSX 19 kb)

Supplementary material 2 (XLSX 13 kb)

Supplementary material 3 (XLSX 15 kb)
